# A lab-on-phone instrument with varifocal microscope via a liquid-actuated aspheric lens (LAL)

**DOI:** 10.1371/journal.pone.0179389

**Published:** 2017-06-26

**Authors:** Yiin-Kuen Fuh, Zheng-Hong Lai, Li-Han Kau, Hung-Jui Huang

**Affiliations:** 1 Institute of Opto-mechatronics Engineering, National Central University, Jhongli City, Taoyuan County, Taiwan; 2 Department of Mechanical Engineering, National Central University, Jhongli City, Taoyuan County, Taiwan; The Ohio State University, UNITED STATES

## Abstract

In this paper, we introduce a novel concept of liquid-actuated aspheric lens (LAL) with a built-in aspheric polydimethylsiloxane lens (APL) to enable the design of compact optical systems with varifocal microscopic imaging. The varifocal lens module consists of a sandwiched structures such as 3d printed syringe pump functionally serves as liquid controller. Other key components include two acrylic cylinders, a rigid separator, a APL/membrane composite (APLMC) embedded PDMS membrane. In functional operation, the fluidic controller was driven to control the pressure difference and ALPMC deformation. The focal length can be changed through the pressure difference. This is achieved by the adjustment of volume change of injected liquid such that a widely tunable focal length. The proposed LAL can transform to 3 modes: microscopic mode (APLMC only), convex-concave mode and biconcave mode. It is noticeable that LAL in the operation of microscopic mode is tunable in focus via the actuation of APLMC (focal length is from 4.3 to 2.3 mm and magnification 50X) and can rival the images quality of commercial microscopes. A new lab-on-phone device is economically feasible and functionally versatile to offer a great potential in the point of care applications.

## Introduction

Mimicking the human eye with ciliary muscles [1, 2], adaptive and focus tunable lenses are of great commercial value in facilitating the low cost, and highly efficient compact optical systems without any physical displacement of the lenses. Several industrial applications were reported previously included reconfigurable dielectric liquid droplet [3], optical beam control [4] and optical zoom [5]. Varifocal endoscopy with piezoelectric actuator [6] and smartphone enabled microscope [7–9].

Two driving methods are predominantly used in the applications of variable focus lenses. First involves the usage of fluidic pressure to sequentially changing the elastomeric membrane of lens radius of curvature. The second method is to use two immiscible liquid interfaces such that different applied voltage can change the meniscus radius of curvature and the resultant focal length. Polydimethylsiloxane (PDMS) thin film is the typical material for the elastomeric membrane and fluid actuated pressure can be used to obtain various focal lengths in the optical system [10, 11]. The gravity-induced differential can be eliminated by matching the fluids densities [12, 13]. An elastomeric array of micro-fluidic network with a multiple of tunable micro-lens was realized via pressure regulating chamber [6, 14]. Other actuation mechanism of variable-focus optical lens systems includes electromagnetic actuators [15, 16], voltage-induced electro-wetting meniscus [17, 18], stimuli-responsive hydrogels [19], photo-polymer [20] and liquid crystal rotation [21–24].

On the other hand, aspherical lenses are widely used and have the advantage of aberration correction of performance [25]. In particular, aspherical lenses are very efficient in correcting spherical coma and longitudinal spherical aberration (LSA) [26], distortion in wide-angle lenses of camera [27], intraocular lens (IOL) [28, 29] and contact lenses or glasses design [30]. Furthermore, by integrating the aspheric lenses with adaptive lenses, spherical aberration can be minimized [31]. The mass-manufacture and typical aspheric PDMS lens (APL) can be fabricated using the gravity-driven hanging droplet technique [32–35, 9]. Furthermore, the additional benefit of a tunable meniscus-like layer to counter for spherical aberrations had been reported and at the optimal thickness ratio of 1:4, the measured spherical aberration can be achieved of—0.023mm, or 56% improvement compared with 1:1 thickness ratio of—0.053μm [36]

This paper describes a dual-function lens with varifocal microscopic modes via the liquid-actuated aspheric lens, which is fully embedded in a smartphone camera in a portable and versatile functionality. A facile, fast and low cost fabrication process is proposed for producing such a varifocal lens, which with superior imaging performance, called liquid-actuated aspheric lens (LAL), which is composed of tunable fluidic APL/membrane composite (APLMC) and aspheric polydimethylsiloxane lens (APL).

The paper will be structured in the following sections. In sections 1, we describe the process of LAL fabrication. First, fabricate APL with method of hanging droplets which allow LAL to microscopic mode with varifocal and has magnification 50x. Next, 3 modes of operations are demonstrated as microscopic mode, convex-concave mode, and bi-concave mode. In sections 2, a detailed optical properties of LAL were measured in terms of the tenability of focal length, contact angle and fringe visibility. In sections 3, comparative investigations of the images captured by the LAL embedded smartphone camera was made against a commercial microscope, and demonstrating tunable imaging properties of proposed device. We envision a new generation of adaptive opto-fluidic devices can be widely adopted and implemented in any smartphone such that with superior optical performance and pervasive lab-on-phone can be easily realized.

## Fabrication of liquid-actuated aspheric lens (LAL)

**Fig 1:**
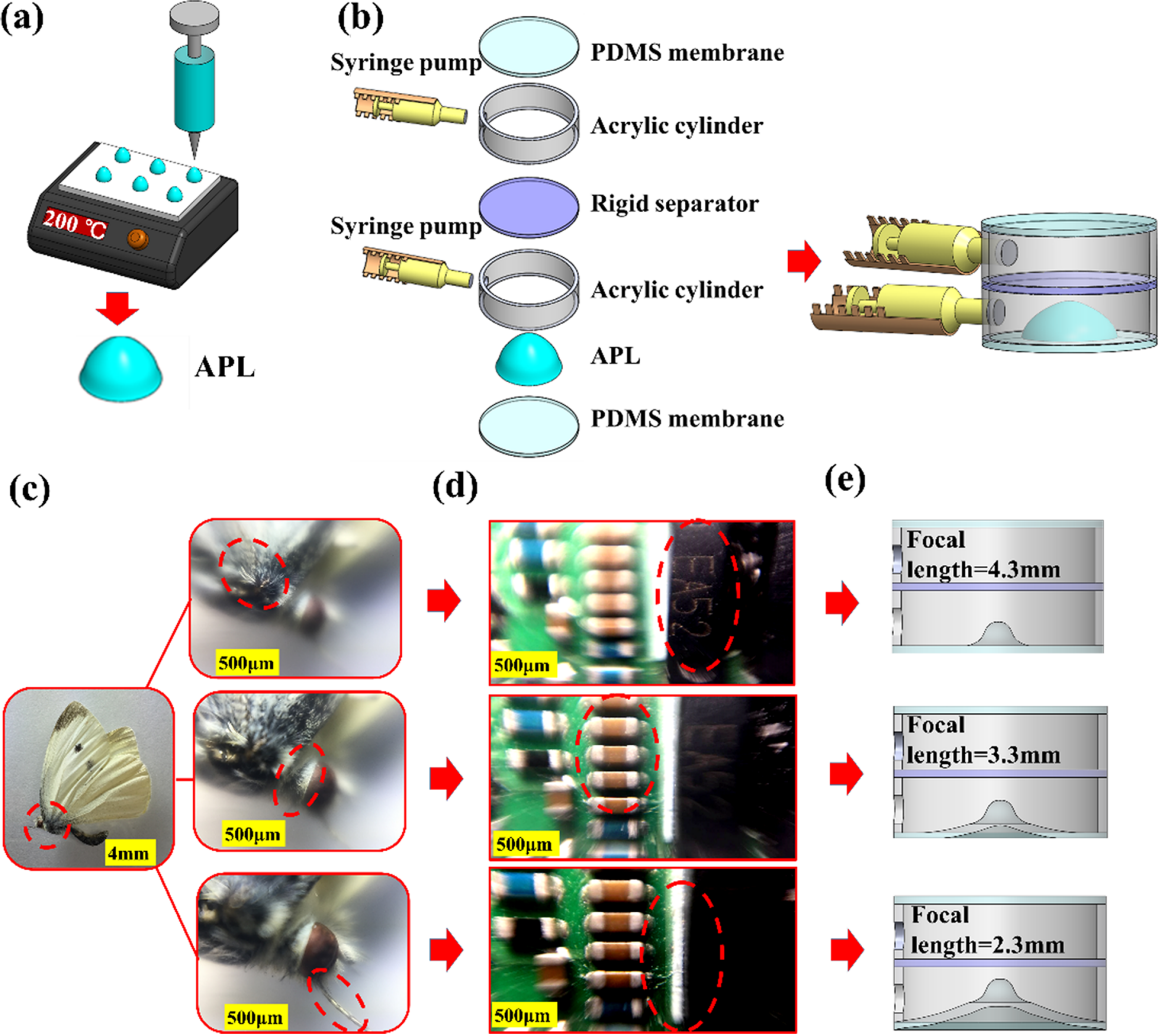
Schematics of polydimethylsiloxane (PDMS) lens fabrication process via heat-assisted approach as illustrated (a) by controlling both preheat platform surface temperature and the volume of the PDMS droplet, thermally curing and stabilizing by heating ~200°C and 30 sec. (b) Schematic for the construction of LAL with an exploded diagram, showing the key components of deformable PDMS membrane, acrylic cylinder, syringe pump, rigid separator (glass slide, refractive index 1.523) and aspheric PDMS lens membrane composite (APLMC). Demonstration of microscopic mode with varifocal capability in the focal length tunability of 2.3–4.3mm for (c) the biological characteristics of wings, mouthparts and compound eyes of Eurema hecabe. (d) microelectronic components of the printed circuit board. (e) Schematically illustrated for 3 types of microscopic mode, focal length is in the range of 4.3 mm to 2.3 mm.

The fabrication process of liquid-actuated aspheric lens (LAL) is the following steps. Aspheric polydimethylsiloxane (PDMS, n = 1.4) lens (APL) is a thermally curable elastomer and functionally applicable to the fields of shorter focal lengths and higher magnification [21]. PDMS solution (2 parts—Sylgard 184, Dow Corning) was prepared by mixing manufacture recommended proportions of PDMS base and curing agent by a weight ratio of 10:1. After mixing and vacuum bubble removal, LAL can be fabricated. The main driving force for actuating a microscopically smooth and curved surface lies on the minimal interfacial surface energies, which automatically trigger the adjustment of optimal contact angle, which can be thermally intervened by accelerated/decelerated curing in situ. The fabricated APL as shown in Fig 1(A) has a significant surface curvature with dimension of 2.1 mm height and 4.2 mm diameter, which is structurally robust and subsequently fabricated by rapid heating to prevent excess spreading. Two key processing parameters are the preheated surface temperature and the volume of the droplet, which predominately controls the focal length via the droplet’s surface curvature. The surface curvature of PDMS droplet and the focal length can be reliably controlled by the balance of gravitational and capillary forces.

Detail construction of LAL with both explosive and assembly views can be illustrated in Fig 1(B). A crucial component of the LAL is an aspheric PDMS lens membrane composite (APLMC), which can be liquid-actuated to change the focal length. The fabrication process of PDMS membrane is similar to aforementioned APL [7, 8] and the fabricated thickness ~500 μm. The top and bottom fluidic chamber serves as a rigid frame and made by acrylic cylinder (1 mm thick) which has the diameter and height of 10 mm and 3.5 mm, respectively. An integrated syringe pump is 3-dimensional (3d) printed and connected with tapped hole to provide volumetric changes. Details pertaining to the 3d-printed syringes, material, acrylic cylinder and specification of 3d printer are presented in the Fig A & B & C in S1 File. In addition, the uniformity of the fabricated PDMS membrane has been experimentally measured and present in the Fig D in S1 File, showing the consistent repeatability. Other key components include the rigid separator portion of the LAL which is consists of circle glass (12 mm diameter and 1 mm thick, Menzel-Glaser, Superfost, refractive index 1.523). The top and bottom portion of the LAL are consists of PDMS membrane and APLMC, respectively. In addition, a transparent liquid of fixed volume (microscope oil, n = 1.515~1.517) and a rigid frame (acrylic cylinder) are both integral parts of proposed LAL. As shown in the assembled and explosive view, both chambers can be individually controlled to trigger the deformation of PDMS membrane as well as APLMC.

**Fig 2:**
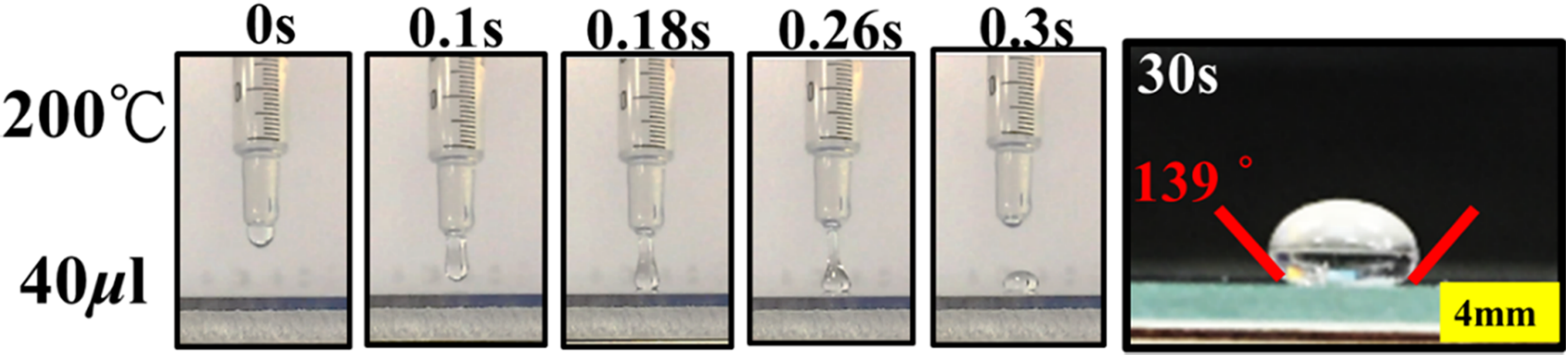
Evolution of PDMS droplet morphology cured at 200° and time sequence of 0–0.3 s. Side view of the APL base on the volume of PDMS solution (40μl) and the contact angles is measured as ~139°. Scale bar is 4 mm.

Functionally speaking, LAL achieved a wide range of focal length tunability via three distinctive operation modes (a microscopic mode: 2.3–4.3mm, convex-concave mode: 1.2–2.5cm and bi-concave mode: 6.2–62.5cm). In general, the focal length tenability can be accomplished in any one of aforementioned mode by the combined adjustment of APLMC dimension, the refractive index of the microscope oil and the extent of the bulging of APLMC. The focal length of the LAL can be designed to decrease or increase depending on injected volume (microscope oil), i.e., as the negatively or positively actuated radius of curvature membrane, respectively. In actual demonstration of microscopic mode with varifocal capability, Fig 1(C) and 1(D) are experimentally show the focal length tunability in the range of 2.3–4.3mm. For the biomedical application, Fig 1(C) shows the biological observations for the wings (top, focus 2.3mm), compound eyes (middle, focus 3.3mm) and mouthparts (bottom, focus 4.3mm) of Eurema hecabe. In comparison, Fig 1(D) shows the optical images on the microelectronic components of PCB board, taken with the same LAL at the microscope mode. Three distinctive focus points can be clearly identified as the alphabetical letters of EA52 (top), orange color capacitors (the size 0.5×1mm^2^, middle) and the white Legend (0.5mm width, bottom), which correspond to the images at different topography at various focal depths. The working mechanism for 3 types of microscopic focal length in the range of 4.3 mm to 2.3 mm, are schematically illustrated in Fig 2(E). The varifocal capability is primarily realized through the liquid-driven actuation of APLMC and resultant movement of light path/axis.

Previously cured at lower temperatures ~70°C, PDMS droplets require a longer time (~1hr) to thermally cure the viscous liquid across the surface by balancing the equilibrium of gravity- pulled liquid surface. [7, 8]. In this paper, an accelerated and heat-assisted curing is proposed. In addition, a smaller diameter can be successfully achieved (7.32 mm v.s 4 mm in diameter) and the resultant significant surface curvature can be attained to induce effective for refractive ray bending. Fig 2 shows the morphological evolution of 40 μL droplets during in situ curing at 200°C. At 200°C, the PDMS droplet virtually cures on contact, causing significant curvature, the contact angle and fabricated lens diameter are measured as ~3.9 mm and 139°. In comparison, the similar contact angle of 138°, bigger lens diameter ~5mm and longer curing time (~90 sec. due to larger PDMS droplets volume, 50 μL v.s. 40 μL) had been reported [9]. In general, both heat-assisted curing processes achieved no burning or charring defects as observed at these temperatures.

### 1. Operation principle and optical qualities

**Fig 3:**
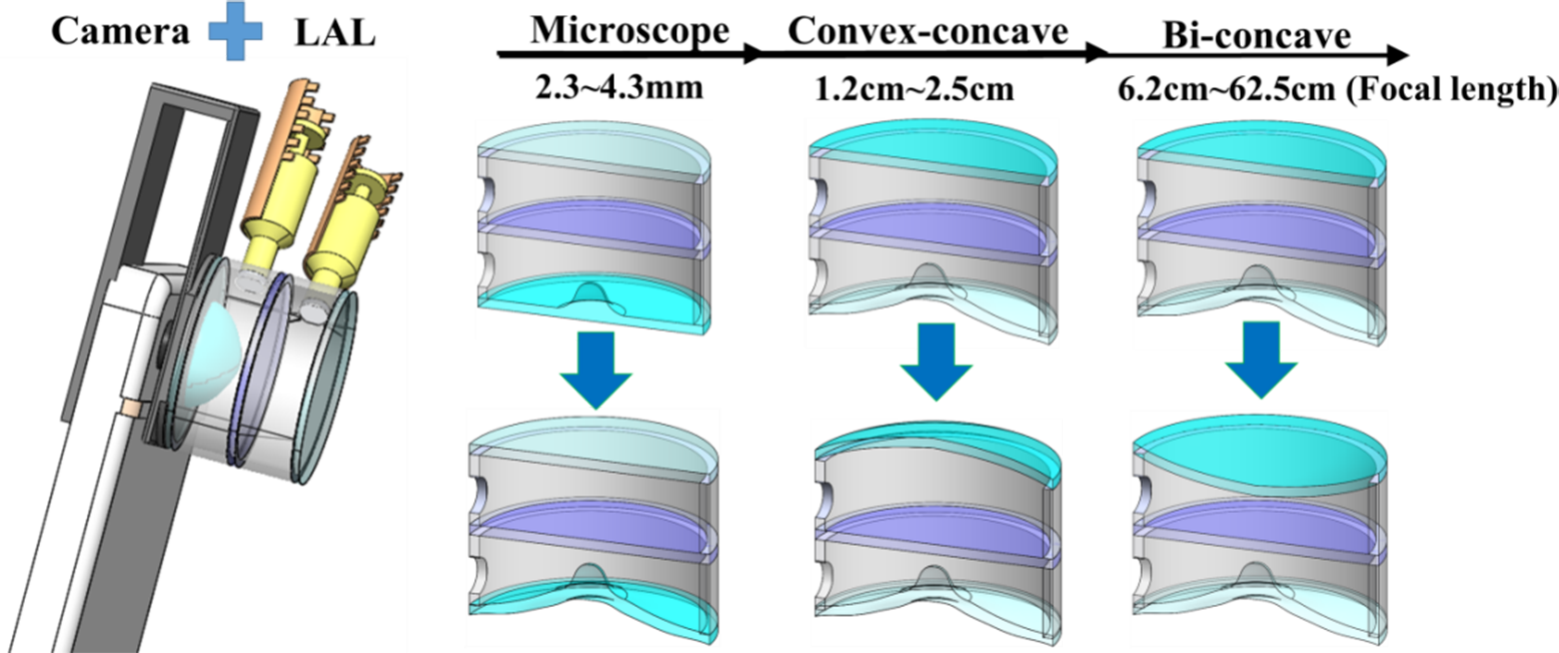
The structure and working mechanism of proposed LAL. Three distinctively different modes can be operated as microscope, convex-concave and bi-concave modes, respectively. The proposed LAL can be easily mounted on a smart phone via a 3d printed fixture as indicated. In the microscope mode, the tunable shapes of APLMC vary with injected volume at the bottom chamber (tunable range is experimentally measured 2.3~4.3 mm). For the operations of convex-concave mode and bi-concave mode, the tunable range can be achieved as 1.2~2.5cm (macro mode) and 6.2~62.5cm (macro mode) respectively.

Fig 3 shows a cross-sectional schematic of the structure and working mechanism of proposed LAL. Three distinctively different modes can be operated as microscope, convex-concave and bi-concave modes, respectively. The tunable microscope mode is achieved via the displacement of APLMC, which the focal length is measured in the range of 2.3~4.3mm. The combination of both convex-concave mode and bi-concave modes can achieve the commercial use of macro mode, which the focal length is in the range of 15cm~50cm. The proposed convex-concave and bi-concave mode can comfortably function as the macro mode, which the focal length tenability is in the range of 1.2~2.5cm and 6.2~62.5cm, respectively. Also shown in the schematic, 3 modes operation is primarily accomplished through the combination of APLMC (lower portion) and PDMS membrane (upper portion) shapes change. Working individually and/or simultaneously, the significant focus length changes can be achieved from 2.3mm to 62.5cm. It is concluded that the focus can be consistently tuned and agrees well with the experimental results for the APLMC/PDMS membrane deformations under applied pressure.

**Fig 4:**
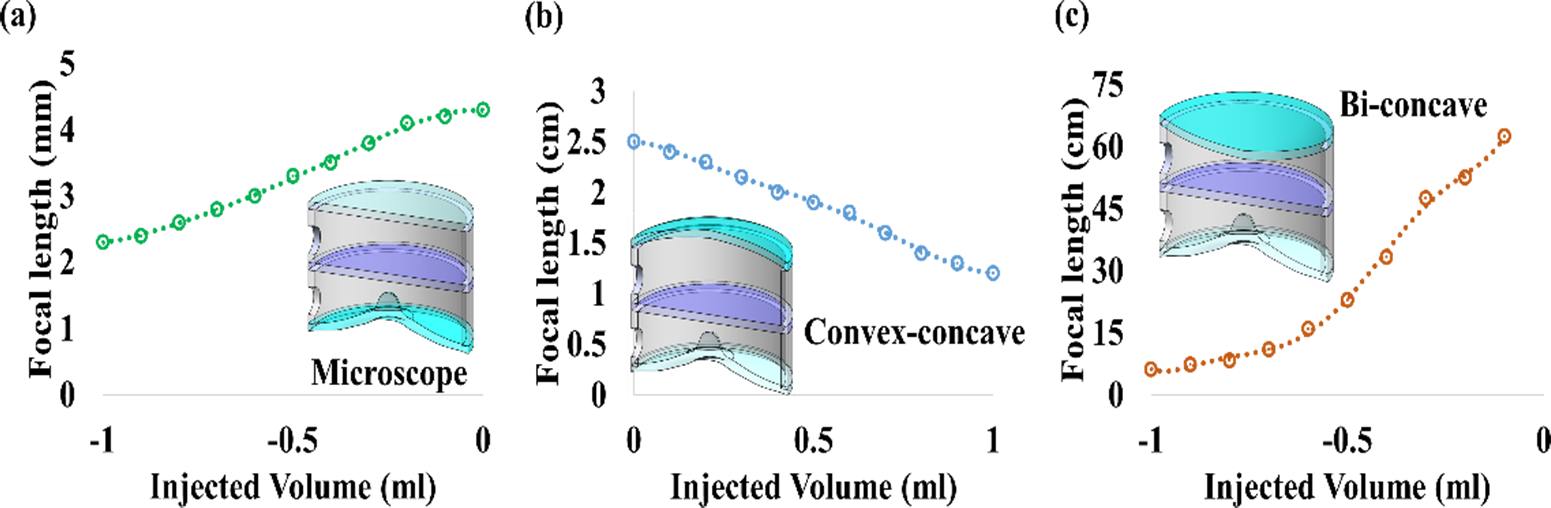
Measured focal length for 3 distinctive operation modes of microscopic mode (APLMC only), convex-concave mode, and bi-concave mode, respectively, (a) Measured microscope mode focal length as a function of the injected volume for APLMC without microscope oil, the focal length of LAL contained wide range in the microscopic mode from 4.3 mm to 2.3 mm (magnification 50X). (b)As a function of the injected volume for microscope oil liquids, measured convex-concave mode focal length from 1.2mm to 2.5 mm.(c) For the operations of bi-concave mode(injected volume for microscope oil liquids), the tunable range can be achieved as 6.2~62.5cm.

The optical qualities of proposed LAL operates at three different modes (microscope mode, convex-concave and bi-concave) is experimentally measured and plotted as focal lengths for three mode types as a function of the injected volume as presented in Fig 4. As shown in Fig 4(A)–4(C), the focal length of the LAL is adjustable by precisely control the injected fluidic volume. In addition, an integrated syringe pump is 3d printed and connected with tapped hole to provide volumetric changes. By continually increasing/decreasing the volume of fluid in the reservoir to compress/stretch the APLMC/PDMS membrane in the configurations of concave/convex respectively, a variable focal length can be varied continuously. Fig 4(A) shows the result in microscope mode, which the measured focal length change is measured to be 4.3 mm to about 2.3 mm with the magnification 50X (for top fluidic chamber is fixed and bottom fluidic chamber injected volume 0/-1 ml, microscope mode). Fig 4(B) shows the operation mechanism in convex-concave mode, the microscope oil is initially filled with both top and bottom chambers to form a pair of fluidic lenses, The focal length change is measured to be 1.2 cm to~2.5 cm (for top /bottom fluidic chamber injected volume is 0.1/0.1 to 1ml/-1ml, convex-concave mode). Similarly, Fig 4(C) shows the working mechanism of bi-concave mode, the measured focal length change is 2.5cm to ~62.5 cm (for top /bottom fluidic chamber injected volume is 0.1/0.1 to -1ml/-1ml, bi-concave mode). In addition, the relationship of injected volume can be experimentally related to the internally applied lens pressure and focal length, as presented in Fig E in S1 File.

### 2. Image performance

**Fig 5:**
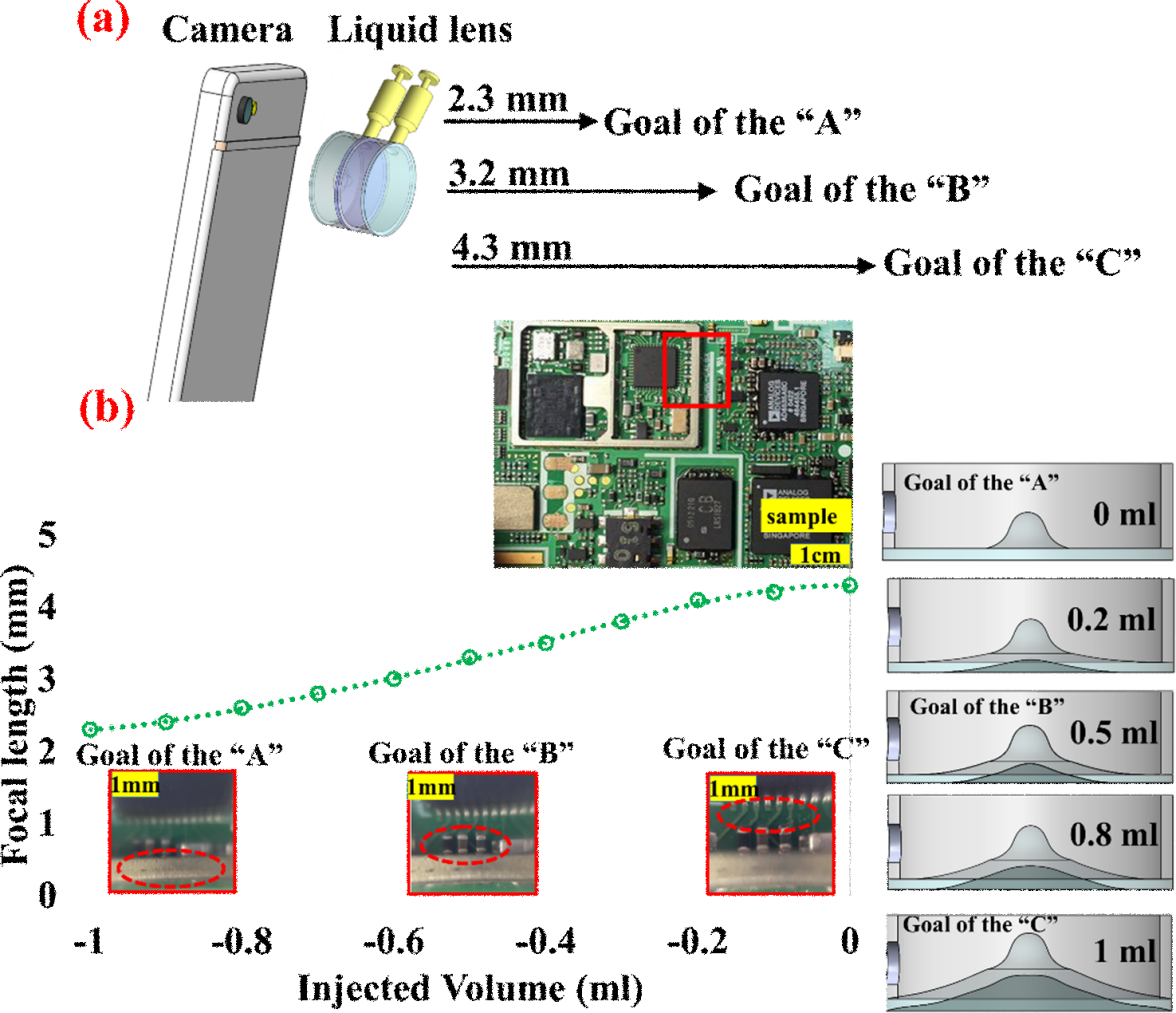
(a) Implementation and experimental setup of the LAL embedded smart phone camera and detailed characterization of the variable focal length (2.3~4.3mm) in the microscopic mode. (b) Schematics of working mechanism (right) of LAL with differential increment of injected volume (0~1 ml) in microscopic mode. Photographic images of PCB can be captured as shown schematically (left) from the LAL at 3 different focuses.

The ability of the LAL (microscope mode) to distinguish three objects positioned at various focus by changing APLMC was successfully demonstrated, as shown in Fig 5. Fig 5(A) shows three depth of focus, the “A” sized 1.5×3mm^2^, the “B” sized 0.5x1mm^2^, the “C” sized 0.5×0.5mm^2^ (each of them was printed circuit board at various positions) were used as the objects. They were purposely positioned 2.3 mm, 3.2 mm and 4.3 mm away from the LAL, and Fig 5(B) (right) shows the working mechanism of LAL, taken with LAL at the microscope mode, respectively. The LAL initially focused the "metal sheets" of PCB as shown in Fig 5(B) (left). To focus the "black capacitors (the size 0.5×1mm^2^, middle)" a fluid was applied to the LAL system. When the injected fluid was decreased to 0.5 ml, “metal sheets” became blurred and “black capacitors” became clearer. When the injected fluid was decreased to 1 ml, “metal sheets” and "black capacitors" became blurred and “green Legend” became clearer. Through the fluid-actuated APLMC, the shift of the focal length can be achieved. All the captured images were realized via a smartphone camera and the proposed LAL device. Above functional versatility and focal length tunability of microscope mode can be easily accomplished by simply changing the fluid volume in the LAL.

**Fig 6:**
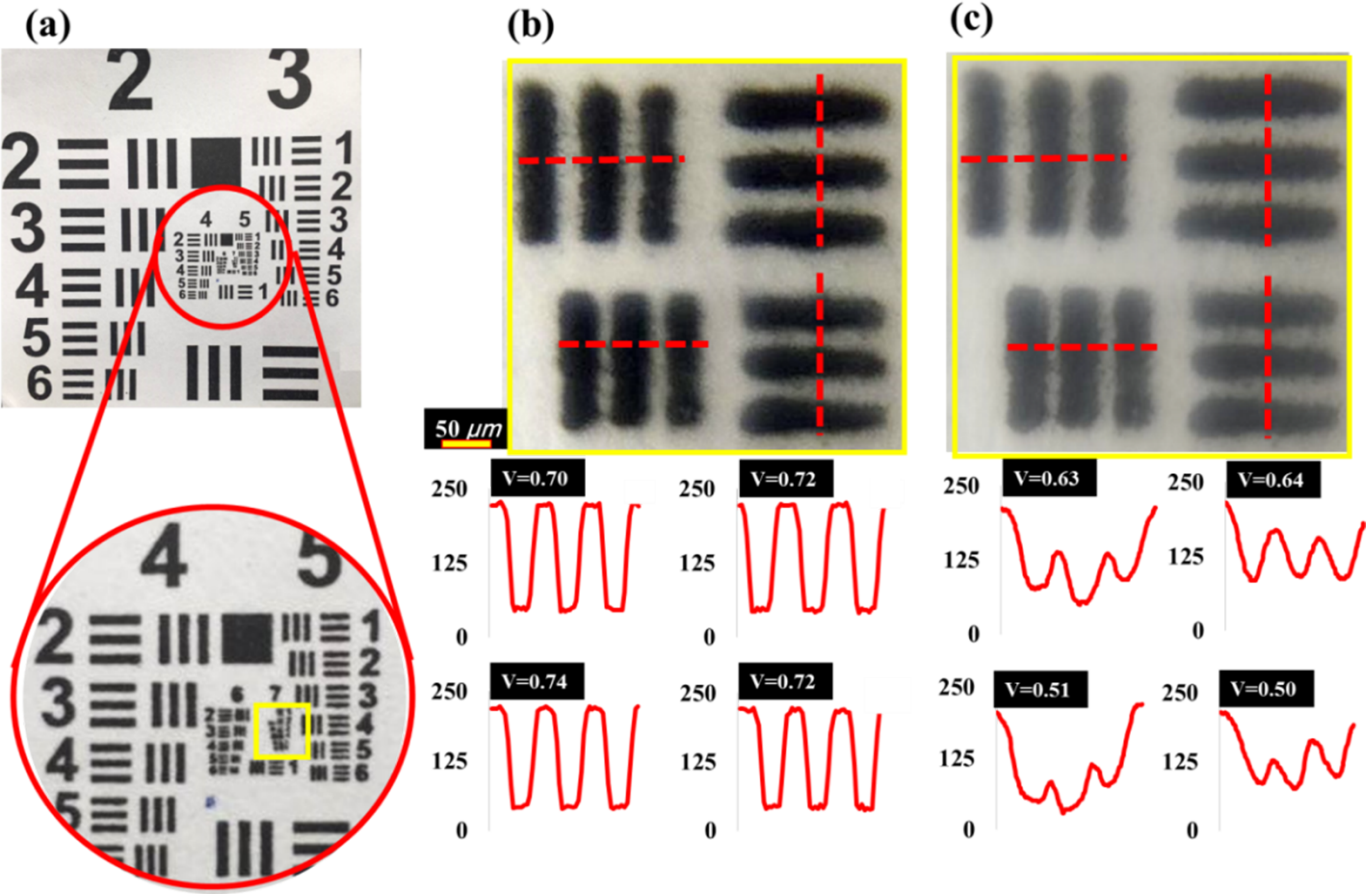
(a) resolution test target images captured through the LAL embedded smartphone camera; a USAF 1951 resolution card with line widths of 55.68 μm (top patterns) and 49.61 μm (bottom patterns) was used for the minimum resolvable feature size measurement; (b) images obtained with 200× microscope and (c) obtained with a I-Phone 6 smartphone with a 40 μl, 200°C APL, operating in the microscope mode. Scale bar is 50 μm.

Fig 6(A) shows the resolution test target images as captured through the smartphone (I-Phone 6). The central portion of the target was imaged with a commercial microscope (20 × 0.40 NA, Nikon, LE Plan) microscope with 200× magnification, as shown in Fig 6(B). The image obtained by the smartphone via the proposed LAL system is shown in Fig 6(C). The USAF 1951 patterns selected are from group number 3 element 2 vertical and horizontal (top), and group 3 element 3 vertical and horizontal (bottom), yielding a respective line width of 55.68 and 49.61 μm. The image has been transformed into gray scale, and intensity profiles of the images were obtained by MATLAB^®^, with the highest intensity at 256 bits and the lowest intensity of 0 bits. Visibility (V) contrast of interference with the optical system can be calculated as (I max–I min ∕ I max +I min). An ideal and featureless output yields a visibility of 1 and 0. The visibility measurement for each USAF 1951 test pattern is summarized and presented in Table 1. It is seen that the visibility of the 55.68 and 49.61μm test patterns from the smartphone-LAL remains lower than that of the commercial microscope. The commercial microscope demonstrates a uniform fringe visibility in the range 0.7–0.74, while the proposed smartphone-LAL demonstrates fringe visibility of 0.5–0.64. However, the intensity image clearly displays the two peak patterns. In comparison, other additive manufactured lens system achieved fringe visibility of 0.18–0.30, which is comparatively unfavorable to the proposed LAL device. Overall, the proposed smartphone-LAL system are optically comparable to those of the commercial microscope, in terms of USAF 1951 target, which the visibility and minimum resolvable feature can be evaluated as a compact and low cost alternative.

**Table 1:** Fringe visibility and intensity of USAF 1951 resolution card image.

Imaged with	Microscope(200X)				Smartphone LAL (Microscope mode,200X)			
**Linewidth**	55.68	55.68	49.61	49.61	55.68	55.68	49.61	49.61
**I-max**	228	255	225	255	223	225	221	220
**I-min**	39	40	38	43	50	48	71	73
**Fringe** **visibility**	0.70	0.72	0.74	0.72	0.63	0.64	0.51	0.50

**Fig 7:**
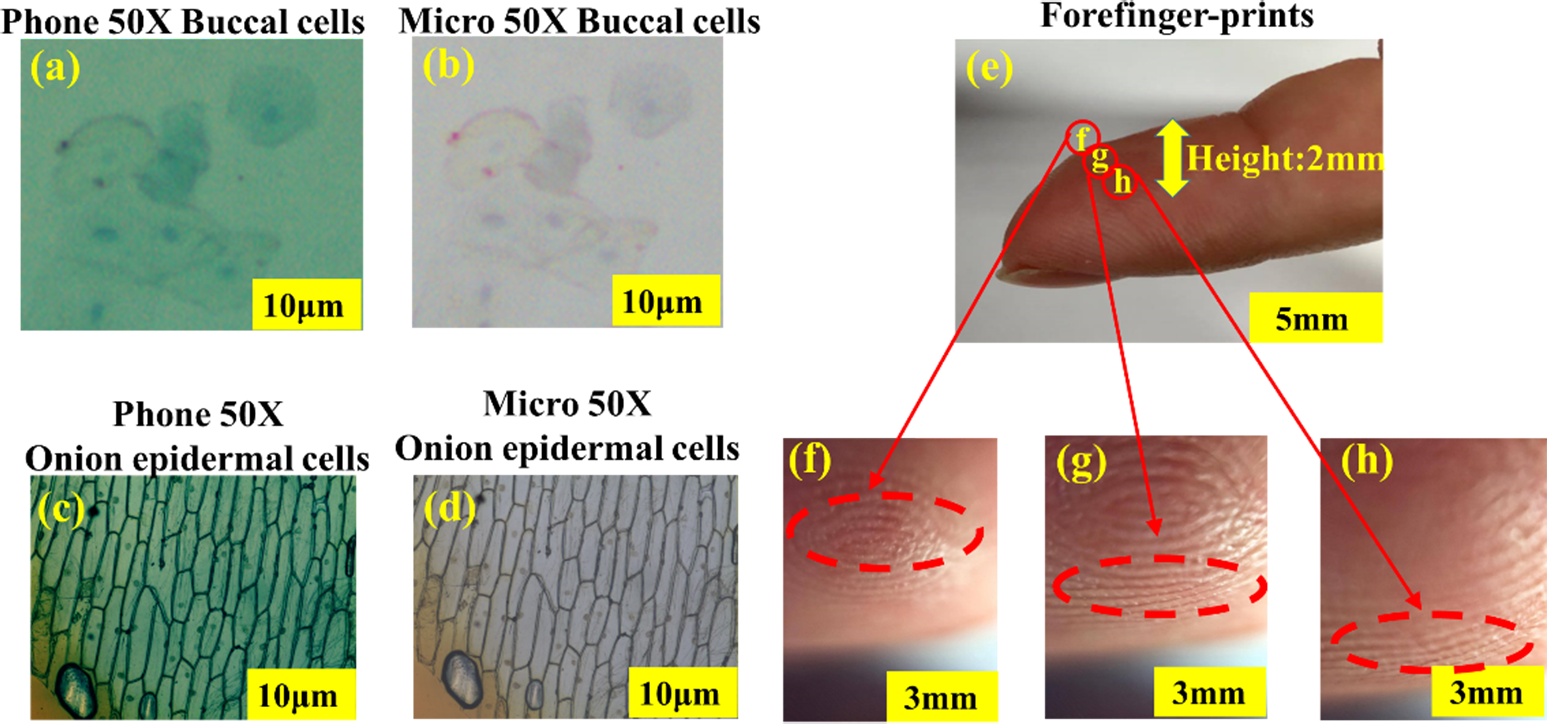
Biological observations of human buccal cells and onion epidermal cells. (a), (c) show the magnified regions, respectively. (b), (d) are the same images captured with a commercial microscope (10 × eyepiece and 5 × 0.75 NA objective lens) with 50× magnification, and with an I-Phone 6 smartphone with LAL (microscope mode). (e) Imaging a human fingerprint that has out of plane features at three depth of focus. The images taken with LAL (microscope mode, corresponding to magnification 20X) of forefinger-prints and the top, middle and bottom row correspond to the images at various depths of 2.3–4.3mm, comparison of the images with 3 tunable focal length in (f), (g), (h), respectively. Scale bar: (a), (b), (c), (d) 10μm; (e) 5mm; (f), (g), (h) 3mm.

We qualitatively compare the performance of the LAL (microscope mode, corresponding to magnification 20X/50X) imaging with a commercial microscope (10 × eyepiece and 5 × 0.75 NA objective lens). We use human buccal cells, onion epidermal cells and forefinger-prints as biological samples for comparison. Similarly for comparison purpose, imaging on a commercially available microscope system (Microtech M 1000) is also performed. The images of human buccal cells and onion epidermal cells taken with the commercial microscope are shown in Fig 7 (B) and 7(D), and the same samples taken with the LAL (microscope mode) are also shown in Fig 7(A) and 7(C) respectively. The images taken by the LAL embedded smartphone camera (microscope mode) here are shown to be equivalently comparable to existing commercial microscopes. Furthermore, optical images taken in Fig 7(A)–7(D) correspond to images by immersion of methylene blue staining liquid, yield to better image quality and features such as buccal cells nucleus and the onion epidermal wall cells can be clearly identified. Fig 7(E) schematically demonstrates the varifocal microscopic imaging of a human forefinger-print (corresponding to magnification 20X) to bring the center fingerprints into focus (2.3–4.3mm focus). By the adjustment of injected volume, the focal length tenability of 2 mm can be achieved. Fig 7(F) and 7(G) obtained with an I-Phone 6 smartphone with a 20μl of APLMC, the top, middle and bottom rows correspond to the images at varifocal depths of 2.3–4.3mm, respectively. The images acquired by the varifocal smartphone microscope are optically comparable with that by a commercial microscope. The LAL-based microscope systems are much smaller and structurally compact than commercial microscopes such that mobile and low cost lab-on-phone can be easily implemented to carry and observe micro and biological structures outdoors. The demonstrated results offer a great potential of high quality optical imaging in a pervasive environment and non-laboratory setting.

**Fig 8:**
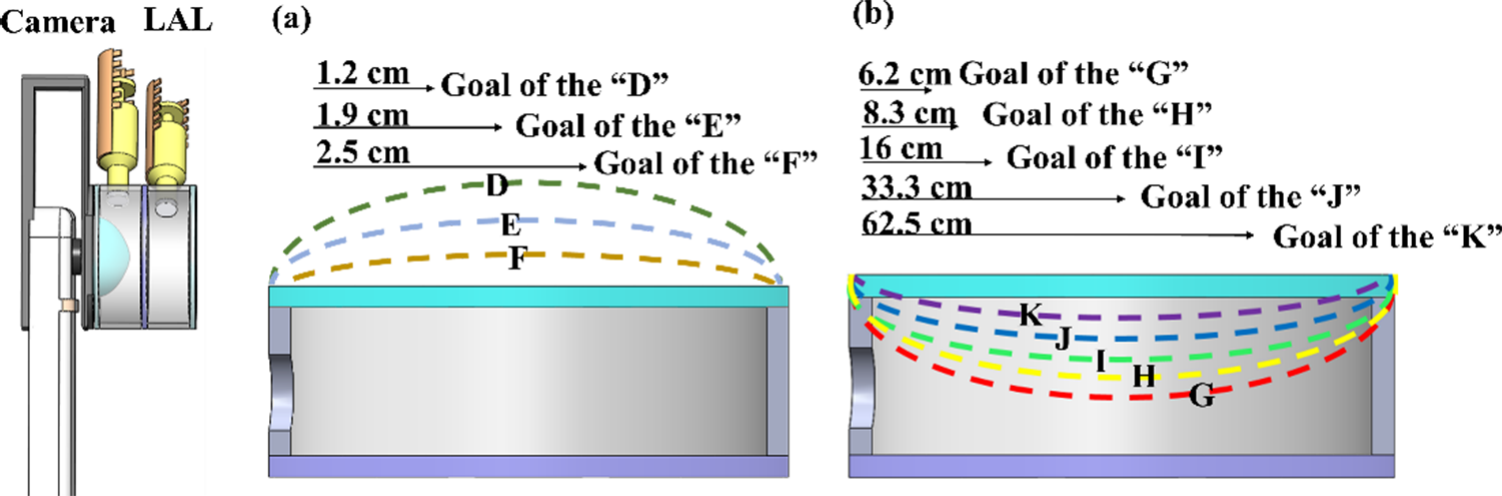
The deformation states of LAL at different focal lengths and the operation modes of convex-concave and bi-concave. (a) Schematics of LAL with various injected volume in convex-concave mode with the variable focal length (1.2cm~2.5cm) (b) variable focal capability in the bi-concave mode, five goals of various focal lengths in the range of 6.2cm~62.5cm. Commercial camera lenses possess macro-mode typically have focal lengths in the range of 15-50cm.

Fig 8(A) shows the schematics of convex-concave mode and optical performance between the various injected fluid volumes and the resultant PDMS membrane variations. The tea green, light blue and straw yellow curves represent the convex-concave mode of the membrane shapes as well as corresponding image planes. Three goals of various focal lengths in the range of 1.2 cm~2.5 cm are demonstrated. It was 1.2 cm distance from the “D” to variable focus LAL. It was 1.9 cm distance from the “LAL” to the “E”. It was 2.5 cm distance from the “LAL” to the “F”. Similarly in the bi-concave mode, Fig 8(B) shows the schematics of between the injected various fluid volumes and the resultant PDMS membrane variations. It was shown that five goals of various focal length in the range of 6.2 cm~62.5 cm. The red, yellow, green, blue and purple curves represent the bi-concave mode of the membrane shapes as well as corresponding image planes. Overall, the proposed LAL is capable of tuning very large range of focal length in two operation modes and compared favorably with the commercial camera lenses possess macro-mode typically have focal lengths in the range of 15-50cm.

**Fig 9:**
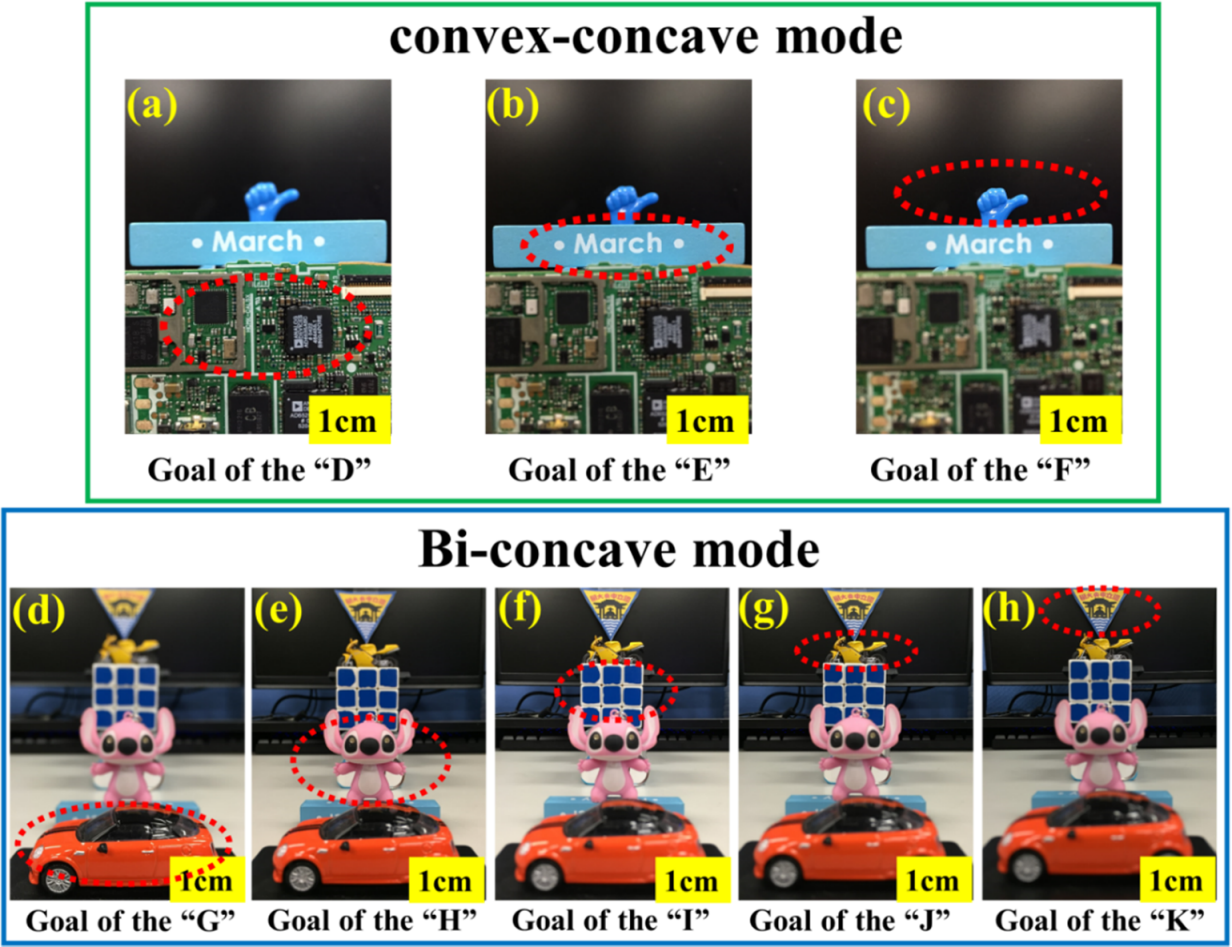
Photographic images at various focal lengths as captured by a smartphone CCD image sensor. (a) The focal length is 1.2 cm, (b) 1.9 cm and (c) 2.5 cm, respectively in the mode of concave-convex; similarly in the bi-convex mode to tune the focal length for (d) 6.2 cm, (e)8.3 cm, (f) 16 cm (g) 33.3 cm and (h) 62.5 cm, respectively. Scale bar: 1cm.

In order to demonstrate the focusing capability of the proposed lens, Fig 9 shows the images taken from the smartphone camera at different focal length, in the operation mode of concave-convex.There were three objects at convex-concave mode, where “D” is the “printed circuit board”, the “E” is “March” word version, the “F” is “thumb-up model”. When focused on the “D” as shown in Fig 9(A), the image was clear while other two images of the “E” in Fig 9(B) and “F” in Fig 9(C) were fuzzy. Adjusting individually the injected volume of top fluidic chamber and bottom fluidic chamber as 0.5 ml and -0.5 ml, the image of the “E” was clear and the images of the “D” and “F” were fuzzy. Similarly, the image of the “F” in Fig 9(C) was in focus as the top fluidic chamber and bottom fluidic chamber injected volume was regulated as 1 ml and -1 ml. Images of the “D” and “E” were out-of-focus. On the other hand for the operation of bi-concave mode, Fig 9(D)~9(H) show the photographs for the images quality at five different focal lengths. For example, Fig 9D–9H show the captured images of fabricated LAL on the object of (d) car model, (e) Stitch model, (f) rubik's Cube, (g) motorcycle model and (h) NCU logo with tunable focal length in the range from 6.2 cm to 62.5 cm. In Fig 9, we carefully control injected volume (When the top fluidic chamber and bottom fluidic chamber injected volume was 1 ml and -1 ml) For example, the NCU logo located at 62.5cm focal length shows a clear image clear as shown in Fig 9(H). At this focal length, other images shown in Fig 9D–9G) become blurrier, particularly on the edge of captured images It is noted that tunable range of the absolute values of the focal lengths of both operation modes cover a wide range (6.2 cm to about 62.5 cm) while the variation of focal length is consistently match the injected volume.

### 3. Conclusion

In this paper, a facile and simple fabrication is proposed and in particular, a compact, tunable and portable microscope is embedded on a smartphone via the elastomer-liquid actuated system. A new paradigm of lab-on-phone can be realized such that a structurally compact and functionally equivalent to the commercial counterpart can be achieved at very low cost. The proposed LAL built using this concept yield relatively large focal length changes (2.3mm-62.5cm) in three different operation modes (microscopic mode, convex-concave mode and bi-concave mode). Comparing with the commercial lenses of macro mode operation, the proposed structure of LAL shows a favorable focal tunability for convex/bi-concave modes from 1.2/6.2 cm to about 2.5/62.5 cm without any mechanical moving components. Moreover, when LAL switches to microscopic mode, the tunable focal length can be varied from 4.3 mm to 2.3 mm at the magnification 50X. Using these LAL, a commercially available smart-phone camera can be easily transformed into a low cost-effective, portable and focal length tunable digital microscope that small structures such as human buccal cells, printed circuit board, onion epidermal cells and forefinger-print can be readily visualized. A new paradigm of lab-on-phone with equivalent image quality of commercial microscopes can be readily accessed, which strongly facilitate the progress of point of care diagnostics and on chip inspections.

## Supporting information

S1 File **Fig A.** Schematic drawing of 3d-printed syringe. Details dimensions pertaining to the 3d-printed syringes are specified above with the following physical dimensions: length: 72.13mm, outside diameter (OD): 5.56mm, nozzle length: 8.13mm, nozzle OD: ∅4.35mm, nozzle inside diameter (ID): ∅1.50mm. Material: Polypropylene (PP)**. Fig B.** Schematic drawing of the acrylic cylinders (red rectangle) and detail dimension is the followings (OD: ∅12.95mm, ID: ∅ 10.04mm, height: 5.95mm). **Fig C.** Optical photo of the used 3D printer (SmartBot S2., http://smartbot.com.tw/product.html). Printing dimension: 200 x 210 x 420 mm. Nozzle Size: 0.4mm. Nozzle Heat: 180–245°C. Heated Bed Temperature: 50–120°C. Point Accuracy: X-Y Resolution 0.01mm; Z Resolution 0.00125mm. Minimum Layer Height: 0.038m. Consumptive Material: PLA, ABS. Filament Diameter: ∅1.75mm. **Fig D.** (a) Uniformity of the PDMS membrane (Diameter: ∅12.951mm, thick: AVG: 0.373mm, standard deviation: 0.002mm) (b) Measurement procedure as performed by the surface profiler (DetakXT, BRUKER.) **Fig E.** (a) Relationship of applied lens pressure versus injected volume (b) Relationship of applied lens pressure versus focal length.
(DOCX)
