# Supplementary material for: A lab-on-phone instrument with varifocal microscope via a liquid-actuated aspheric lens (LAL)
Source: PLoS One. 2017 Jun 26;12(6):e0179389. doi: 10.1371/journal.pone.0179389 (PMC5484483; doi:10.1371/journal.pone.0179389)
Supplement: S1 File — Fig A. Schematic drawing of 3d-printed syringe. Details dimensions pertaining to the 3d-printed syringes are specified above with the following physical dimensions: length: 72.13mm, outside diameter (OD): 5.56mm, nozzle length: 8.13mm, nozzle OD: ∅4.35mm, nozzle inside diameter (ID): ∅1.50mm. Material: Polypropylene (PP). Fig B. Schematic drawing of the acrylic cylinders (red rectangle) and detail dimension is the followings (OD: ∅12.95mm, ID: ∅ 10.04mm, height: 5.95mm). Fig C. Optical photo of the used 3D printer (SmartBot S2., http://smartbot.com.tw/product.html). Printing dimension: 200 x 210 x 420 mm. Nozzle Size: 0.4mm. Nozzle Heat: 180–245°C. Heated Bed Temperature: 50–120°C. Point Accuracy: X-Y Resolution 0.01mm; Z Resolution 0.00125mm. Minimum Layer Height: 0.038m. Consumptive Material: PLA, ABS. Filament Diameter: ∅1.75mm. Fig D. (a) Uniformity of the PDMS membrane (Diameter: ∅12.951mm, thick: AVG: 0.373mm, standard deviation: 0.002mm) (b) Measurement procedure as performed by the surface profiler (DetakXT, BRUKER.) Fig E. (a) Relationship of applied lens pressure versus injected volume (b) Relationship of applied lens pressure versus focal length. (DOCX) [file pone.0179389.s001.docx]

**Supporting Information**

Of the manuscript “A lab-on-phone instrument with varifocal microscope via a liquid-actuated aspheric lens (LAL)”


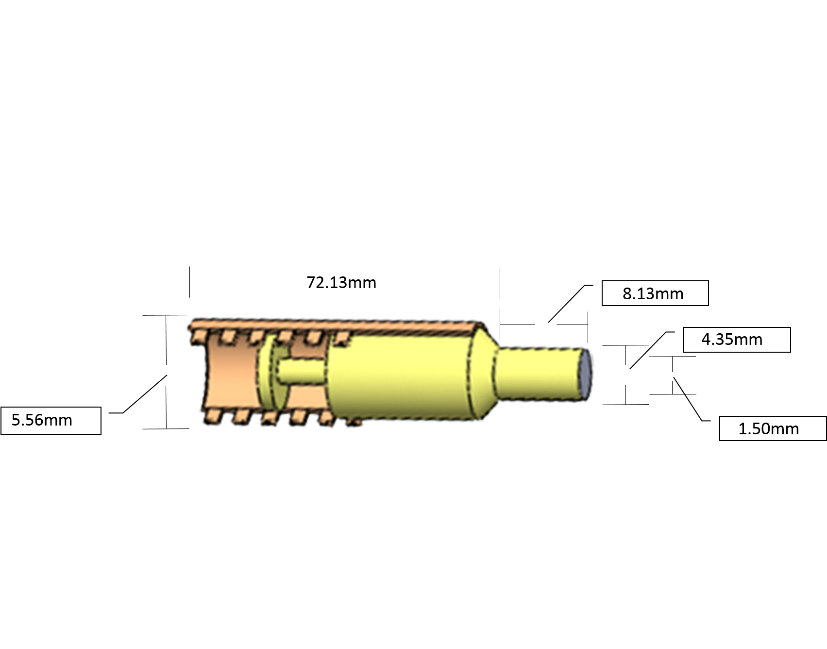

Fig A. Schematic drawing of 3d-printed syringe. Details dimensions pertaining to the 3d-printed syringes are specified above with the following physical dimensions : length: 72.13mm, outside diameter (OD): 5.56mm, nozzle length :8.13mm, nozzle OD: $\emptyset$4.35mm, nozzle inside diameter (ID):$\emptyset$1.50mm. Material: Polypropylene (PP)


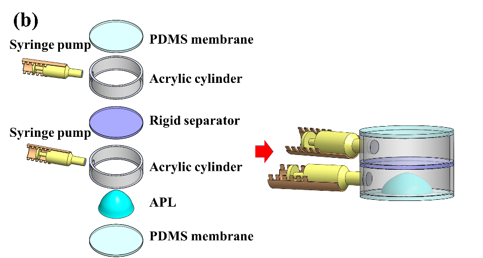


Fig B. Schematic drawing of the acrylic cylinders (red rectangle) and detail dimension is the followings (OD : $\emptyset$12.95mm, ID :$\emptyset$ 10.04mm, height : 5.95mm )


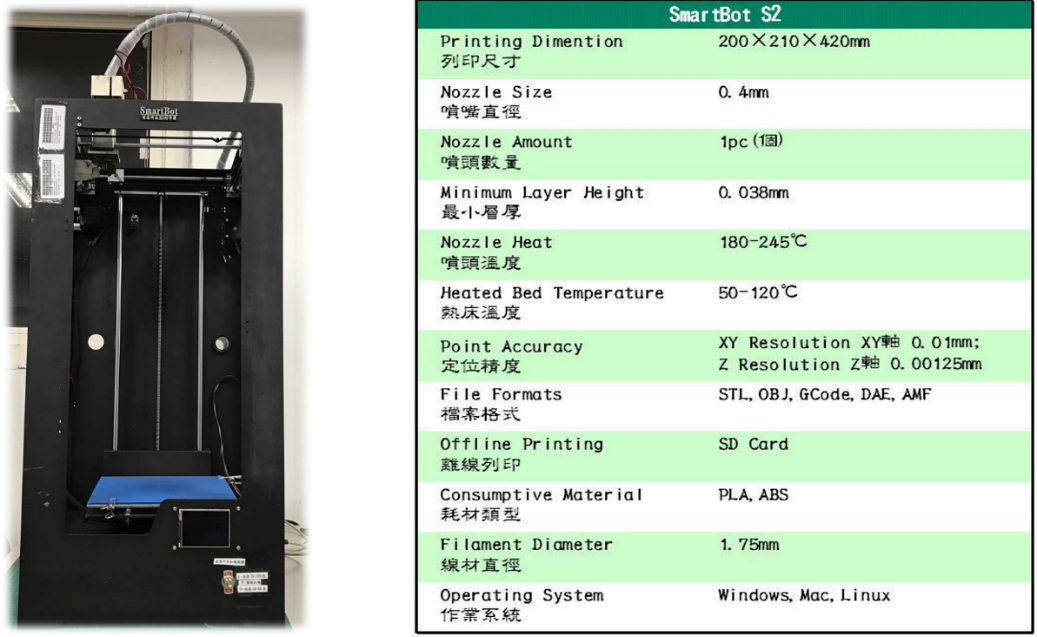


Fig C. Optical photo of the used 3D printer (SmartBot S2., http://smartbot.com.tw/product.html). Printing dimension : 200 x 210 x 420 mm. Nozzle Size : 0.4mm. Nozzle Heat: 180-245 $℃$. Heated Bed Temperature: 50 - 120$℃$. Point Accuracy: X-Y Resolution 0.01mm ; Z Resolution 0.00125mm. Minimum Layer Height: 0.038m. Consumptive Material: PLA, ABS. Filament Diameter : $\emptyset$1.75mm.

(a)


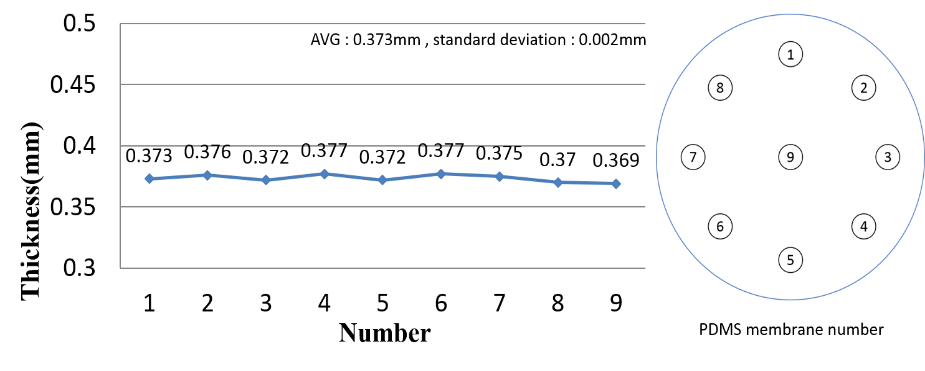


(b)


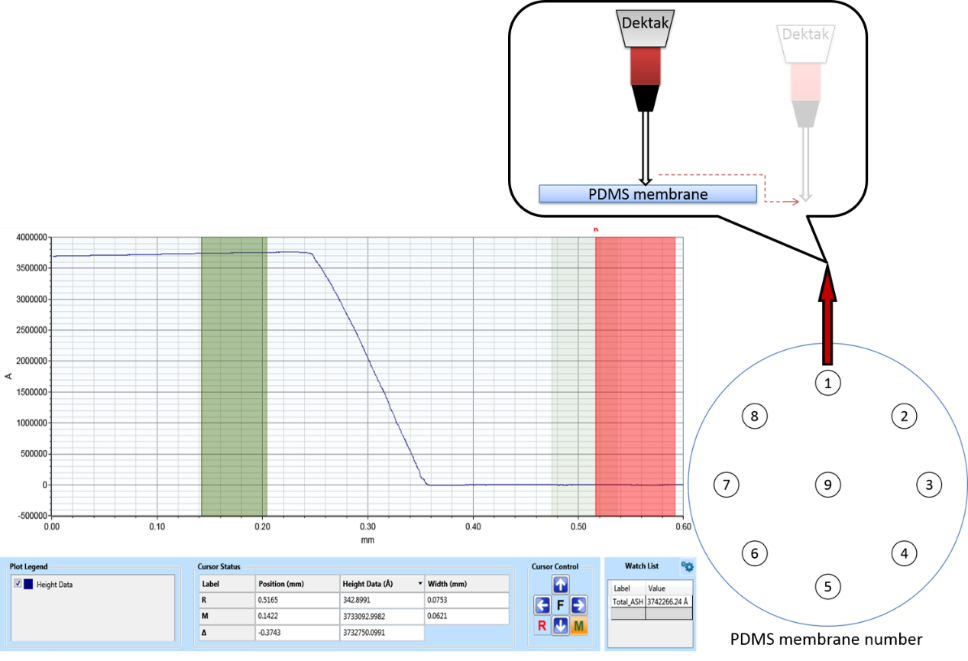


Fig D. (a)Uniformity of the PDMS membrane (Diameter : $\emptyset$12.951mm, thick : AVG : 0.373mm , standard deviation : 0.002mm) (b) Measurement procedure as performed by the surface profiler (DetakXT, BRUKER.)

(a)


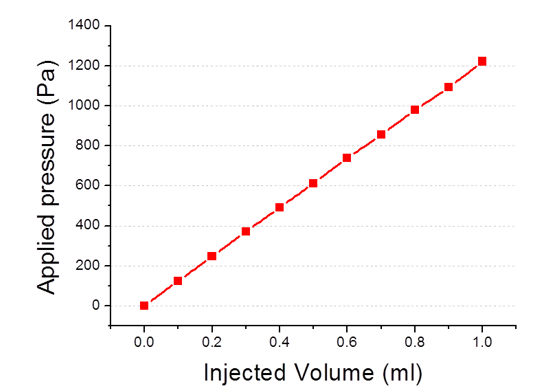


(b)


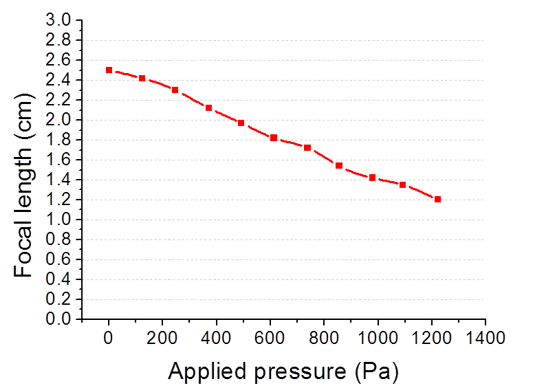


Fig E. (a) Relationship of applied lens pressure versus injected volume (b) Relationship of applied lens pressure versus focal length.

Table S1 Original data of Fig. 5

|  | Injected Volume (ml) | Focal length (mm) |
| --- | --- | --- |
| 1 | -1 | 2.3 |
| 2 | -0.9 | 2.4 |
| 3 | -0.8 | 2.5 |
| 4 | -0.7 | 2.7 |
| 5 | -0.6 | 2.9 |
| 6 | -0.5 | 3.2 |
| 7 | -0.4 | 3.5 |
| 8 | -0.3 | 3.8 |
| 9 | -0.2 | 4 |
| 10 | -0.1 | 4.2 |
| 11 | 0 | 4.3 |
